# Share capitalism and worker wellbeing^⋆, ⋆⋆^

**DOI:** 10.1016/j.labeco.2016.09.002

**Published:** 2016-09-14

**Authors:** Alex Bryson, Andrew E. Clark, Richard B. Freeman, Colin P. Green

**Affiliations:** a UCL, NIESR, and IZA, United Kingdom; b Paris School of Economics, CNRS and IZA, France; c Harvard, NBER, and CEP, United States; d Lancaster University, United Kingdom

**Keywords:** Job satisfaction, Wages, Compensation methods, Working conditions, J28, J33, J54, J63, J81, M52

## Abstract

We show that worker wellbeing is determined not only by the amount of compensation workers receive but also by how compensation is determined. While previous theoretical and empirical work has often been preoccupied with individual performance-related pay, we find that the receipt of a range of group-performance schemes (profit shares, group bonuses and share ownership) is associated with higher job satisfaction. This holds conditional on wage levels, so that pay methods are associated with greater job satisfaction in addition to that coming from higher wages. We use a variety of methods to control for unobserved individual and job-specific characteristics. We suggest that half of the share-capitalism effect is accounted for by employees reciprocating for the “gift”; we also show that share capitalism helps dampen the negative wellbeing effects of what we typically think of as “bad” aspects of job quality.

## 1. Introduction

In the absence of detailed information on job attributes, measures of worker wellbeing, and in particular job satisfaction, have been proposed as a potential summary metric of overall job quality (Hamermesh, 2001). There are now a number of contributions that have addressed the validity of such subjective measures (see Clark et al., 2008; De Neve et al., 2013). In the cross-section, these have been shown to predict future objective outcomes, such as life-expectancy and health in general, marriage, divorce and fertility. In the specific context of the labour market, job satisfaction scores predict future job quits (Clark, 2001; Green, 2010) and retirement (Clark et al., 2015), and wellbeing has been linked to greater productivity at work (Oswald et al., 2015). None of these results would be found were subjective scores not to be comparable to at least a certain extent across individuals.

The use of these types of stated-preference measures has improved our understanding of how workers respond to changes in job characteristics and contractual arrangements. One empirical regularity is that wages, unsurprisingly, are positively correlated with job satisfaction. A more recent literature has sought to examine whether the way in which wages are determined, and in particular performance-pay schemes that explicitly link compensation to effort and output, also influence job satisfaction. As discussed below, this literature has largely focused on individual performance-pay schemes. However, group-based performance-pay schemes appear to be at least as common as individual-based performance pay in Europe and the United States (Bryson et al., 2013). This is the subject of the current paper, which provides a range of evidence that performance pay, and specifically group-payment schemes, has a robust positive impact on job satisfaction.

In standard theoretical models there is a clear connection between individual performance-based pay and worker wellbeing. The linking of pay to individual performance aims to compensate workers for the disutility of effort by setting marginal product and rewards equal. Performance pay hence allows workers to choose the effort and pay combination that maximises their utility (Lazear, 1995). In practice, this may not occur for a number of reasons. These include workers lacking the job autonomy to influence output, or the employer setting the effort–reward ratio to the disadvantage of the worker. In these cases, it has been suggested that performance pay may actually result in worse worker wellbeing outcomes, including lower morale, greater stress and anxiety, injury, and absenteeism (Bender et al., 2012; Frick et al., 2013).

The effect of group-based payment schemes on worker wellbeing has attracted less attention, despite these schemes being relatively common. The theoretical link between group-based performance pay and worker utility is less clear. For instance, the incentive-based channels discussed above are likely diluted due to the 1/n problem. It has been argued, however, that what can be described as ‘Share Capitalist’ modes of pay (broadly, in which worker pay depends on the firm’s fortunes) potentially affect worker wellbeing through a variety of alternative channels.

Employees with a direct financial stake in the firm, for instance where pay is linked to firm profits, may feel more engaged in the decision-making process within the organization. And even when this ownership or profit-sharing stake is modest, the firm’s promotion of such schemes may perform what Bowen and Ostroff (2004: 206) describe as “a symbolic or signalling function” to communicate a strong HRM system that is capable of aligning the interests of the organization and the worker. A second potential channel is that the provision of workers with an ownership stake in the firm may be viewed as a form of gift exchange. Along these lines, Bryson and Freeman (2014) argue that standard all-employee share-purchase plans are a ‘gift’ from the employer, since they offer discounted shares, often by giving workers free shares for every share they buy, up to a limit. This may increase worker wellbeing through the ‘warm glow’ created by this gift. This may be related to the value of the gift, but even small value payments may increase wellbeing as they have been shown to influence worker performance (Kosfeld and Neckermann, 2011). Additional, less direct, transmission channels also exist. For instance, it is possible that the high take-up of a share plan among co-workers positively affect non-members’ wellbeing. Non-members may like having reciprocating types of co-workers, especially in the case of a positive production externality.

However, it is not guaranteed that group-based payment will increase job satisfaction. One key criticism of the effectiveness of group-based payment relates to free-riding. In practice, these problems may not manifest themselves due to increased co-worker peer pressure and co-monitoring (Kandel and Lazear, 1992), as has been shown in recent empirical work (Freeman et al., 2010). This is akin to the effects of what Barker (1993) termed the ‘concertive control’ exercised in teams. Whilst this may be good for the company, a culture of worker co-monitoring focused on encouraging greater worker effort has potentially detrimental effects on worker motivation and job satisfaction (Green and Heywood, 2010). In addition, group-based payment, in common with performance pay more generally, exposes workers to greater earnings risk, which may also be associated with lower wellbeing (Cornelissen et al., 2011).

Group-based payment may then influence worker wellbeing in a variety of ways, including a number that are distinct from the channels proposed for individual performance-related pay schemes. The main focus of the current paper is to provide estimates of the effect of group-based payment schemes on job satisfaction in three distinct data settings: a single-firm (ShareCo), European cross-sections (EWCS), and a British panel (the British Household Panel Survey: BHPS). These settings are complementary insofar as they allow us to disentangle the specific forms of group-based payment schemes from other performance-pay schemes, and to examine their effect on worker wellbeing in narrow within-job settings. Our approach is to use these three datasets to establish a credible body of evidence on: (1) the effect of performance-pay schemes on job satisfaction; (2) the way in which performance pay influences worker dissatisfaction with poor working conditions; and (3) the spillover effect of performance pay on non-recipients’ job satisfaction.

Our main result is that group-based schemes are robustly positively correlated with job satisfaction in all three datasets, and across different specifications. By way of contrast, and as a matter of interest, this is not the case for individual performance-payment schemes. We go on to explore two possible channels for which our data is well-suited. First, we examine the potential role of worker reciprocity by focusing on organisational loyalty and perceptions of fairness, both of which may be influenced directly by performance-related pay. The loyalty channel may especially hold for pay methods such as profit-sharing and share receipt, where one purpose is to make workers ‘part-owners’ of the firm and so view it is a joint enterprise. Second, we ask whether workers in group-payment schemes report smaller falls in wellbeing when exposed to negative employment conditions. We posit that group-incentive schemes may dampen the negative impact of poor working conditions on employee wellbeing, via increased loyalty to the firm or a feeling of firm ownership in share-capitalist schemes.

The remainder of the paper is organised as follows. Section 2 presents the existing empirical evidence, and Section 3 describes our data and empirical approach. Section 4 presents the results and Section 5 then concludes.

## 2. Empirical evidence

Kruse et al. (2010: 262) review 12 contributions in the area, and conclude that the evidence on performance pay and worker wellbeing is at best mixed. We start by highlighting two papers that are most closely related to our work here.

Green and Heywood (2008) use the BHPS to provide panel data estimates (1998–2004) of the effect of performance pay on job satisfaction. Their focus is on individual performance pay, but they also provide estimates for profit-related pay/bonuses. They find the latter are associated with higher job satisfaction, in both cross-section and panel regressions. Our analysis of BHPS builds on their work by extending the period of analysis (1998–2008), conditioning on a broader array of work characteristics, and focusing on group-based performance pay. Reflecting their focus on individual performance pay, the estimation sample in Green and Heywood (2008) includes both private- and public-sector workers. While individual performance pay has become more common in the public sector, it is less clear how many typical forms of group payment would operate in this setting (e.g. the difficulty in defining a surplus/profit to be shared). We focus only on BHPS private-sector workers, where group-based payment is likely to be salient, and condition on worker-job fixed effects, whereas Green and Heywood (2008) confine their analysis to worker fixed effects. Also, we utilise a range of additional variables including payment size that have not previously been analysed and which allow us to paint a more complete picture of the relationship between compensation schemes and job satisfaction.

Kruse et al. (2010) examine the effect of a variety of share-capitalist type compensation schemes on worker outcomes using two US-based data sets: the 2002 and 2006 waves of the General Social Survey (GSS), which is a representative sample of employees at for-profit organisations, and an NBER survey of 14 companies which have at least one group-performance scheme. In the GSS no relationship is found between share-capitalist schemes and job satisfaction. The analogous relationship in the NBER data is positive, but becomes insignificant once company fixed effects are introduced. The same results hold when stock-option and employee-ownership schemes are examined separately. They do however suggest that both profit sharing and gain sharing increase job satisfaction when they are ‘higher-powered’ (i.e. when the scheme’s payment makes up a larger proportion of the worker’s overall compensation).

An alternative to the stated-preference approach of evaluating the correlation between compensation scheme and worker wellbeing is to instead consider ‘revealed-preference’ measures. One such measure is job quitting (see Freeman, 1978; Clark, 2001). Here, the early work of Blakemore et al. (1987) presents a model predicting that bonuses reduce quits and finds empirical support for it in Panel Survey of Income Dynamics data; Lakhani (1988) also shows that re-enlistment bonuses reduced quit rates in the US Army. More recently Bryson and Freeman (2014) use the single-firm ShareCo data (which we also use here) to reveal a negative correlation between share-plan participation and quit intentions. Kruse et al. (2012) confirm that more high-powered share-capitalist schemes reduce voluntary turnover and increase the intention to stay, even among the ‘100 Best Companies to Work For in America’, where we might expect little variation. The explanation appears to be a positive association between share capitalism and the quality of working life, as indicated by more trusting relations with supervisors, greater participation in decision making and more information sharing, which go to make up what they term a ‘more positive workplace culture’.

More broadly, there is a great deal of work suggesting that higher pay increases satisfaction at work (Bryson et al., 2012) and a substantial literature confirming Rosen’s (1974) contention that higher pay can compensate workers for poor work conditions. Poor conditions reduce employee wellbeing (unless they are fully compensated by higher wages or other benefits). Workers with shares in the company may however be less concerned about improving conditions if doing so affects their share prices or the size of their profit share; equally they may identify more closely with the employer than do other employees. As such, we suspect that the job satisfaction of workers with shares will be less sensitive to working conditions than that of other employees.

The following section outlines our empirical approach and describes the three datasets that we use to analyse the relationship between share capitalism and job satisfaction.

## 3. Data and empirical approach

The key challenge in this literature is the identification of the causal effects of performance pay on worker wellbeing. One particular source of concern is unobserved worker heterogeneity with respect to ability, disutility of effort, or preferences for risk or reciprocity. The introduction of performance pay by firms is associated with potential sorting on the basis of a range of factors such as those listed above that are generally not observed in data (Lazear, 1986, 2000). If not accounted for, this sorting may lead to a positive association between performance pay and employee wellbeing. At the same time, workers who face constraints on their ability to choose their preferred compensation package may well be ‘misallocated’. For example, if the number of firms offering shares to employees does not meet employee demand, employees may queue for jobs with share plans, allowing employers to pick from the queue. On the other hand, performance-related pay is standard in some occupations, so that workers in these occupations have little choice but to accept it as part of their compensation package. The ideal experiment to establish the effects of performance pay on worker wellbeing would involve randomly treating individuals, occupations or workplaces within a firm with a particular wage or payment method, or randomly taking a person and moving her to a new firm with a different pay regime. We do not have such experimental data here. Our approach is instead to use three complementary datasets to establish a credible body of evidence on the link between group payment and job satisfaction. Below we set out our underlying empirical approach and describe, in turn, how each dataset is used.

Our basic regression for the relationship between payment methods and job satisfaction is:

(1) $${JS}_{i}=\alpha^{\prime }{\mathrm{PayType}}_{i}+\beta^{\prime }X_{i}+\varepsilon_{i}$$

where *Paytype_i_* is a vector of performance-related payment methods received by worker *i*, *JS_i_* reported job satisfaction and *X_i_* a vector of controls. Job satisfaction has been shown to be a useful predictor of various work-related behaviours, such as quits (Freeman, 1978; Clark et al., 1998; Clark, 2001), absenteeism (Clegg, 1983) and productivity (Mangione and Quinn, 1975; Patterson et al., 1997). As such, it is often considered to be a viable index of the work-related component of utility.^3^ The job satisfaction regressions throughout the paper are estimated using linear techniques, as Ferrer-i-Carbonell and Frijters (2004) suggest that the difference between cardinal and ordinal estimation of subjective wellbeing is not particularly large.

Other things equal we expect performance-pay workers to earn more than their fixed pay counterparts because performance pay compensates workers for additional effort. In the absence of controls for wages, the estimated value of *α* in (1) combines the effect of performance pay, increased wages and job disamenities on job satisfaction. Potentially more interesting is the conditional effect of performance pay holding wages constant, as in the following equation:

(2) $${JS}_{i}=\alpha^{\prime }{\mathrm{PayType}}_{i}+\beta^{\prime }X_{i}+\lambda {\mathrm{Wage}}_{i}+\varepsilon_{i}$$

The estimated value of *α* in Eq. (2) now picks up any effect of performance-related pay that does not come via wages. We estimate pooled and panel variants of these equations in three different data sets. The panel regressions allow any individual differences in response style to be controlled for. As it turns out, all of these different specifications and datasets produce qualitatively very similar findings.

### 3.1. Dataset 1: ShareCo

Our first dataset is single-firm. The company, ShareCo (a pseudonym), is a multinational business services corporation employing roughly 12,000 full-time equivalent employees globally. Our data come from a dedicated web-based survey, designed by two of the authors in conjunction with the firm. We analyse pooled data from this firm in the UK that was collected in 2007 and 2010.

The ShareCo job satisfaction question is: “*How satisfied are you in your job?*”, with responses recorded on a 5-point Likert scale where 1 = very dissatisfied and 5 = very satisfied. Importantly for our purposes, the company operates an employee share purchase plan (ESPP) that is central to its remuneration strategy.^4^ Initially we estimate Eqs. (1) and (2) with the payment methods being share-plan membership and whether the worker is a salaried employee who is also paid bonuses or commissions. We also include a control for the worker’s perceptions of the proportion of employees in the work-unit who belong to the share plan. The *X*’s are a set of individual-level demographic and job characteristics, as listed in the footnote to Table 1.

We then add work-unit fixed effects to each of the above OLS estimates. These units identify groups of employees working in close proximity to each other in that they work in the same office and business division or unit. The within work-unit regressions thus control for any unobserved fixed elements of the working environment that are correlated with both plan participation (and other worker behaviour) and job satisfaction.

### 3.2. Dataset 2: European Working Conditions Survey (EWCS)

Second, we consider the 2000/01 and 2005 waves of the European Working Conditions Survey (EWCS) to see whether various forms of performance pay are correlated with satisfaction with working conditions.^5^ The EWCS surveys roughly 1000 employees per country across 31 European countries, including all member countries of the European Union. Our final estimation sample is 33,510 after dropping observations with missing values on key variables and workers who report that they are single traders (i.e., in an organization where they are the sole worker). We estimate variants of Eq. (2) with the dependent variable coming from the responses to the question “*On the whole, are you very satisfied, satisfied, not very satisfied or not at all satisfied with working conditions in your main paid job?*”^6^ We always introduce country dummies to avoid any issues of the cross-country comparability of ordinal subjective wellbeing measures: we thus present within-country estimates. The EWCS oversamples workers in small countries, but contains detailed weights to adjust for the relative likelihood of workers appearing in the sample. All of our estimations use these weights (although they do not actually affect our qualitative results). In addition to being cross-country, the chief advantage of the EWCS is its information on a number of separate payment schemes, the nature of the job, and its hazards and risks. Below we use EWCS data to see how payment methods are correlated with satisfaction with working conditions, and whether share capitalism mediates the effect of bad working conditions on employee satisfaction. We provide further details below and in the notes to Table 2.

### 3.3. Dataset 3: British Household Panel Survey (BHPS)

Our last dataset is the panel of British employees contained in the BHPS, a general survey covering a random sample of approximately 10,000 individuals in 5500 British households per year, rising to figures of 16,000 and 9000 respectively in later waves. The BHPS is a household panel: all adults in the same household are interviewed separately. We use 11 waves of the BHPS from 1998 to its cessation in this form in 2008, as these years have consistent information on two forms of performance-related pay, as follows:

*In the last 12 months have you received any bonuses such as a Christmas or quarterly bonus, profit-related pay or profit sharing bonus, or an occasional commission?* [this excludes overtime payments]; and *Does your pay include performance-related pay?*

(Taylor et al., 2006)

The answers to these two questions are used in turn to create dummies for bonus/profit-share receipt and other performance-related pay receipt. Those who receive a bonus also report the annual amount, so that we can see whether small contingent payments (such as Christmas bonuses) have only little effect on worker utility compared to higher-powered bonuses. The data also include a wide range of information on individual and household demographics, health, labour-force status, employment and values. There is both entry into and exit from the panel, leading to an unbalanced data.

Our BHPS dependent variable is overall job satisfaction, from the question: “*All things considered, how satisfied or dissatisfied are you with your present job overall using the same 1–7 scale?*” The key advantage of the BHPS is not only its ability to track individual workers over time, but also its job histories data, which allows us to track individuals in specific jobs over time. Following the approach outlined in Green and Heywood (2015), we augment the standard job satisfaction model with worker-job fixed effects. In this way we provide evidence of the effect of performance-related pay on job satisfaction holding both individual and work-specific characteristics constant.

### 3.4. Extensions

Having first estimated the relationship between payment methods and job satisfaction, we then turn to two potential explanatory channels. First, returning to ShareCo, we use information on organisational loyalty and perceptions of fairness, both of which may be directly influenced by performance-related pay. The loyalty channel may especially hold for schemes such as profit sharing and share receipt, where one aim is to make workers ‘part-owners’ of the firm and so view it as a joint enterprise. Equally, perceptions of fair pay may arise if employees believe they are more likely to be paid their marginal product in the presence of performance-pay schemes. Organisational loyalty in ShareCo is measured as the sum of the answers to three questions, all measured on a five-point Likert scale running from “strongly agree” (5) to “strongly disagree” (1): “*I feel very loyal to this organization*”, “*I find that my values and the company’s values are very similar*” and “*Overall this company is a good place to work*”. Our resulting attachment scale runs from 3 (lowest) to 15 (highest), and has a scale-reliability co-efficient of 0.84. The fair-treatment scale is calculated analogously from the answers to “*I am fairly paid relative to my ShareCo colleagues in a similar job*” and “*I am fairly paid relative to employees with similar jobs in other companies*”, and has a reliability coefficient of 0.75.^7^ We add these measures to Eq. (2) to see whether the effect of payment schemes on job satisfaction works via loyalty and fairness.

Our second extension is to ask whether performance pay can mitigate the negative effects of bad working conditions: Do those with poor working conditions react less negatively to them when they also receive bonus and profit-based payments? We first examine this using the wide range of cross-section information in the EWCS. We then turn to BHPS panel data to provide within-job estimates. The BHPS has less information on working conditions, and we here consider two aspects of the job that might realistically be thought to be negative: working unpaid overtime and commuting time.

## 4. Results

### 4.1. Share ownership, profit-related pay and worker wellbeing

We first ask whether workers with shares and other profit-related pay schemes report higher wellbeing. The first row of Table 1 shows that workers who are in the company share plan in ShareCo are more satisfied with their jobs. This finding is robust to the inclusion of work-unit fixed effects, so the result is not driven by fixed unobservable differences across office/business units affecting both satisfaction and the individual’s decision to join the share plan. It is also robust to conditioning on log wages.

Conditional on the individual’s own share plan membership status, ShareCo employees’ job satisfaction also rises with the percentage of their peers who they think belong to the company share plan (Table 1, row 2). The result is robust to the inclusion of work-unit fixed effects. There is thus a positive wellbeing spillover from co-workers’ share ownership. An interaction term between own membership status and perceptions of peers’ membership (not shown) attracted a statistically insignificant estimated coefficient, so the size of the spillover is similar for members and non-members. The implication is that members and non-members value the positive externality they receive from colleagues’ share plan membership to a similar degree. These externalities might include hard working on the part of colleagues, or simply being surrounded by reciprocating types even if you yourself are not one of them. There is no reason to think that non-members would value these sorts of externalities less than their colleagues who were in the plan.

The third row of Table 1 shows that workers with bonuses and commissions, i.e. whose pay is partly tied to results, report higher job satisfaction. However, this effect shrinks and becomes less precise once we condition on work-unit fixed effects. Part of this positive association then reflects work-unit level variation in the use of commissions.

We now estimate similar regressions for the cross-European data over 31 countries in the 2000/2001 and 2005 waves of the EWCS. While we cannot control for work units in the EWCS, we do have highly-detailed information on workplace characteristics, tasks and hazards — many of which are likely to be correlated with the use of performance pay. Table 2 shows the effect of four different, non-mutually exclusive payment types with an increasingly complete control vector. All models incorporate country fixed effects so that we present within-country estimates. Model (1) contains an intercept term and four dummy variables for different types of performance pay, along with income, gender and age. Here there is a negative effect of piece rates on job satisfaction, no effect of group bonuses and positive effects from share payments and profit shares. Model (2) adds a rich array of controls for occupation, industry, tenure, hours, flexible employment contracts and firm size, while model (3) includes controls for autonomy, task type, work hazards, shift work etc., as detailed in the notes to Table 2. These inclusions substantially improve the fit of the model, and the positive relationships between job satisfaction, on the one hand, and profit sharing and share payments on the other continue to hold. However, the estimated coefficient on piece rates becomes insignificant as we add controls, suggesting that the initial negative relationship reflected the type of jobs and working conditions in which piece rates are used.

Last we turn to evidence from the 1998–2008 waves of the BHPS. Table 3 shows the relationship between the receipt of the two types of performance pay in the BHPS, first in pooled regressions and then panel estimates holding within-job and within-worker characteristics constant. As in the EWCS in Table 2, there is no statistically-significant relationship between individual performance pay and job satisfaction. However, bonus receipt and profit sharing are associated with higher job satisfaction, and are robust to the introduction of job-worker fixed effects: for a given worker in a given job, the switch to bonuses/profit shares leads to increased job satisfaction, holding wages constant.

The BHPS data also includes the amount of the bonus/profit share, which we add to the models above. The main motivation for doing so is looking for potential non-linearities in bonus size, such as negative effects of small bonuses on worker behaviour (Gneezy and Rustichini, 2000). In the first two columns of Table 4 the bonus/profit share receipt dummy is replaced by the amount of the bonus: job satisfaction rises with bonus size in both the pooled and within worker-job panel estimates. The quadratic bonus term in column 3 reveals that there is a non-linear effect. To explore further, we re-estimate the model in column 4 with an indicator for small or large bonus payments (greater or less than £1000). While large bonuses are, perhaps naturally, associated with the greatest job satisfaction, there is no evidence of a negative effect of smaller bonuses on worker wellbeing.

To summarise, there is a strong positive association between share-capitalist approaches to payment and job satisfaction. This is true across a variety of institutional settings, including cross-Europe, within firm and across Britain. Moreover, using a variety of data we have demonstrated how this result is robust to approaches that identify within-workplace and within worker-job effects. Taken together, we have a body of evidence that suggests that the introduction of group-based performance-payment methods such as share ownership and profit sharing increase worker wellbeing. In our subsequent analysis we seek to examine, in turn, two possible channels through which group-payment receipt may positively affect worker wellbeing: (1) gift exchange and (2) wage compensation for bad working conditions.

### 4.2. Does gift exchange account for the wellbeing effects of share-plan participation?

Table 5 replicates the ShareCo analysis in the last two columns of Table 1 but now adds controls for organisational loyalty and perceptions of fair pay. We can see if the association between share-plan participation and job satisfaction is driven by more favourable views of the firm, as might be the case if plan participants feel that the plan represents gift-exchange. Wages are insignificant here, but the estimated coefficients on organisational loyalty and perceived fair pay are positive, large and statistically significant: the models here account for roughly two-fifths of the variance in job satisfaction, compared to only around one-tenth in Table 1. Furthermore, the introduction of these new variables reduces the plan membership coefficient by one-half, while the coefficient for the perception of peers’ membership falls markedly. It therefore appears that a considerable part of the plan-membership effect stems from greater organisational loyalty and a heightened perception of pay fairness. Individual plan membership continues to remain statistically significant in most of the models, so that these channels do not account for the entire association.

We also estimate models that include feelings of co-ownership.^8^ The feeling of co-ownership is positively associated with plan membership, and strongly positively correlated with job satisfaction. Its introduction reduces the plan-membership coefficient by roughly half in the job-satisfaction equations. The membership dummy remains statistically significant, albeit only at the 90% confidence level. This continues to be the case when organisational loyalty and perceptions of fair pay are also added to the model, though it becomes statistically non-significant in the fixed effects model. Perceptions of co-ownership thus clearly matter for the job satisfaction of ShareCo employees, and account for a sizeable part of the plan-membership effect, but not all of it.

### 4.3. Do share-capitalist payment methods dampen the negative effects of poor working conditions on employee job satisfaction?

Using the EWCS and BHPS we ask whether tying employee remuneration to firm or group performance makes a difference to the way in which employees respond to bad working conditions.

Table 6 reports EWCS results akin to those in Table 2, but this time splitting the sample into those who receive some form of share-capitalist pay – income from share ownership in their firm, profit-sharing or group-based performance-related pay – and those who do not. The table presents a selection of coefficients related to work conditions which can be considered as unpleasant and are likely to reduce job satisfaction.^9^ While the coefficients are similar for many job characteristics, in general individuals in share-capitalist jobs appear to be more tolerant of a range of negative working conditions.^10^ For instance, share-capitalist workers are not negatively affected by commuting, by having the pace of their work set by the boss, their colleagues, or by targets. There are similar results for having to work to tight deadlines. Share-capitalist workers also have a more muted negative response to threats and discrimination through work. There is essentially no difference in response to health or safety being at risk at work or the number of hazards to which the worker is exposed. These effects emerge despite the fact that the model controls directly for wages. Hence, these results provide some support for the proposition that share-capitalist workers are more forgiving of bad working conditions.

These EWCS results are also found in BHPS panel data, where we consider unpaid overtime hours and travel to work time (minutes). We estimate the negative effect of these two working conditions on job satisfaction, and then introduce interactions to see whether this effect is mitigated by bonus/profit shares receipt. The first two columns of Table 7 refer to pooled OLS estimates. Both unpaid overtime hours and commuting time reduce overall job satisfaction, and in both cases the interaction term with bonus/profit share receipt is positive, although it is only statistically significant in the case of unpaid overtime hours. The size of the estimated coefficients in column 1 implies that unpaid overtime hours significantly reduce satisfaction, but only for those who do not receive incentive payments. Columns 3 and 4 show the results holding worker-job fixed effects constant: these are very similar, although the interaction term between bonus/profit share and commuting time is now statistically significant at the 10% level. In essence, being in receipt of a bonus/profit share appears to substantially mute the negative consequences of these work disamenities on job satisfaction, and this continues to be the case even in our estimates holding job-matches constant. As a result, they do not seem to result from either unobservable worker or job characteristics that jointly influence job satisfaction, working conditions and payment type.

## 5. Conclusion

In this paper we show that worker wellbeing is influenced by how compensation is determined. Those in receipt of group-performance bonuses or profit shares, and those in share-ownership schemes, have higher job satisfaction than do other employees, conditional on their wages. These findings hold across three quite different data sets and are robust to the inclusion of work-unit fixed effects in the ShareCo data, detailed job controls in the EWCS data, and individual fixed effects in the BHPS panel data. In the ShareCo data, the perceived participation of co-workers in the share plan has an additional positive impact on individual job satisfaction, regardless of their own share-plan membership status.

We investigate two channels through which these ‘share capitalist’ modes of pay produce positive worker outcomes. First, in a single-firm setting, we find that about half of the share-capitalism effect can be accounted for by employees’ feelings of reciprocity in return for the ‘gift’ of share capitalism. Second, in broader survey data we show that these payment methods dampen, or in some cases entirely wipe out, the negative wellbeing effects of what we typically think of as bad working conditions.

Although our results are suggestive of a causal link between group performance pay and employee job satisfaction, one which accords with various theories about what might make employees happy, our analyses cannot definitively confirm a causal linkage. We encourage those engaged in field experiments randomly determining employee exposure to different payment systems to collect outcome data relating to employee wellbeing alongside the data on productivity and firm performance that is more commonly collected.

## Supplementary Material

Appendix

## Figures and Tables

**Table 1:** Job satisfaction, share plan membership and bonus commission in ShareCo.

	Without wages		With wages	
	
	OLS	Work-unit fixed effects	OLS	Work-unit fixed effects
Member	0.228*** (0.049)	0.225*** (0.051)	0.235*** (0.050)	0.231*** (0.052)
% member	0.081*** (0.018)	0.085*** (0.019)	0.080*** (0.018)	0.084*** (0.019)
Commission	0.150** (0.062)	0.118* (0.066)	0.144** (0.063)	0.114* (0.067)
Adj. R^2^	0.08	0.09	0.08	0.09

Notes:

(1) The membership dummy is based on the response to the question “*Are you a member of a ShareCo Share Plan?*” The percent membership is based on the following question: “*What percentage of workers in your business unit do you think are members of the ShareCo Share Plan?”* with responses coded 1 = none, 2 = 1–19%, 3 = 20–39%, 4 = 40–59%, 5 = 60–79%, 6 = 80–99% and 7 = 100%. The item is entered linearly. The “Commission” dummy is one of three dummies identifying contractual status, the others being hourly paid and salaried without commission. The “Commission” coefficient is evaluated against the omitted category of “Salaried without bonus/commission”. The regressions contain an intercept and the following controls: age (5 dummies); male; white; degree; professional qualification; household status (4 dummies); sociability scale; risk scale; majority of household income is ShareCo earnings; occupation (7 dummies); supervisory status; hours worked (4 dummies); tenure (5 dummies); and a dummy for the year of the survey. The sociability scale is an additive scale counting the number of times employees ticked a box in response to the following question: “*Do you take part in the following activities, either as part of your job or outside work? Please select as many as apply to you … member of a trade/professional body or association; work in schools, colleges, universities; involved in charities or voluntary bodies; member of a social, sports or arts club; active member of a political party; active member of a religious group; socialising with co-workers outside of work*”. The risk scale is based on responses to the question “*Are you generally a person who is fully prepared to take risks or do you try to avoid taking risks?*” where 1 = “unwilling to take risks” and 10 = “fully prepared to take risks”.

(2) Sample *N* = 1887 without wages and 1846 with wages. The fixed effects models absorb 54 work-unit dummies. Robust standard errors in parentheses. ***, ** and * indicate statistical significance at the 1%, 5% and 10% levels, respectively.

**Table 2:** Satisfaction with working conditions and performance pay, European Working Conditions Survey (EWCS) 2000–2005, private-sector workers.

	(I)	(II)	(III)
Piece rate	−0.094*** (0.020)	−0.061*** (0.021)	−0.014 (0.020)
Profit share	0.073*** (0.028)	0.081*** (0.026)	0.079*** (0.025)
Group bonus	0.026 (0.040)	0.029 (0.041)	−0.024 (0.039)
Share payment	0.103** (0.048)	0.094* (0.050)	0.14*** (0.050)
Observations	33,510	31,113	29,714
Adj. R^2^	0.097	0.126	0.245

Notes:

(1) Robust standard errors in parentheses. ***, ** and * indicate statistical significance at the 1%, 5% and 10% levels, respectively. All estimates adjusted with population weights.

(2) Controls in model (I): an intercept, gender, age, age^2^, income and country (31 dummies).

(3) (II) adds controls for occupation (9 dummies), industry (12 dummies) tenure, hours worked, flexible contract work and firm size.

(4) (III) adds controls for commutes more than 30 min each day, whether there are long hours, whether work pace is set by colleagues, by the machine, by the boss or by targets, worker experiences threats or discrimination at work, health or safety risks at work, number of hazards exposed to at work, shift work, repetitive work, monotonous work, night shift, whether the worker can choose speed of work, order of work, or method of work, presence of quality assessment, problem solving required, telework, homework, complex tasks, task rotation, and the need for learning on the job.

**Table 3:** Incentive payments and job satisfaction, BHPS 1998–2008, private-sector workers.

	(1)	(3)
	
	Pooled OLS	Worker-job match fixed effects
Ln wage (2001£)	0.12*** (0.017)	0.130*** (0.0128)
Performance pay	−0.027 (0.020)	−0.015 (0.017)
Bonus/profit share	0.074*** (0.015)	0.068*** (0.013)
Observations	48,045	48,045
Adj. R^2^	0.050	0.045
Number of worker-job matches		1976

Notes:

(1) Robust standard errors in parentheses. ***, ** and * indicate statistical significance at the 1%, 5% and 10% levels, respectively.

(2) Column (1) includes an intercept and controls for male, age, age^2^, marital status, health status, A-level, diploma, degree or higher, union coverage, large firm (200+), promotion opportunities, employer-funded pension, industry (9 dummies), occupation (9 dummies), and region (11 dummies).

(3) Column (2) omits the time-invariant controls.

**Table 4:** Job satisfaction and bonus size, BHPS 1998–2008.

	(1)	(2)	(3)	(3)
			
	OLS	Worker-match FE	Worker-match FE + bonus^2^	Small & large bonuses
Ln wage (2001£)	0.115*** (0.017)	0.126*** (0.013)	0.120*** (0.013)	0.120*** (0.013)
Performance pay	−0.013(0.019)	−0.004 (0.017)	−0.009 (0.017)	−0.023 (0.017)
Real bonus (£’000 s)	0.005** (0.002)	0.006*** (0.001)	0.013*** (0.001)	
Real bonus^2^ (£1 M)			−0.00002*** (0.000005)	
Bonus < £1000				0.020 (0.015)
Bonus ≥ £1000				0.173*** (0.020)
Observations	48,111	48,111	48,111	48,111
Adj. R^2^	0.050	0.045	0.046	0.046
Number of worker-job matches		1976	1976	1976

Notes:

(1) Robust standard errors in parentheses. ***, ** and * indicate statistical significance at the 1%, 5% and 10% levels, respectively.

(2) The OLS models include an intercept and controls for male, age, age^2^, marital status, health status, A-level, diploma, degree or higher, union coverage, large firm (200+), promotion opportunities, employer-funded pension, industry (9 dummies), occupation (9 dummies), and region (11 dummies). The worker-match FE models (2–4) omit the time-invariant controls.

**Table 5:** Job satisfaction, share-plan membership and bonuses in ShareCo: the role of loyalty and fairness.

	OLS	FE
Member	0.113*** (0.039)	0.105*** (0.042)
% member	0.029** (0.015)	0.032** (0.016)
Commission	0.047 (0.048)	0.052 (0.052)
Log wage	−0.021 (0.017)	−0.021 (0.019)
Loyalty	0.230*** (0.009)	0.228*** (0.009)
Fairness	0.041*** (0.011)	0.040*** (0.012)
Adj. R^2^	0.42	0.42

Notes:

(1) The models contain controls described in the notes to Table 1, and additive scales for organisational commitment and perceptions of fair pay. See the text for details.

(2) Sample N = 1846. Robust standard errors in parentheses. ***, ** and * indicate statistical significance at the 1%, 5% and 10% levels, respectively.

**Table 6:** Impact of “bad” working conditions on satisfaction with working conditions among those with and without share capitalist types of compensation (profit shares or share ownership). EWCS, 2000–2005, private-sector workers.

	(1)	(2)
	
	With share capitalism	Without share capitalism
Commute > 30 min	−0.0366 (0.0363)	−0.0250* (0.0142)
10+ hours at least once per month	0.0507 (0.0499)	−0.0249 (0.0205)
Work to tight deadlines	−0.0391 (0.0419)	−0.0882*** (0.0178)
Pace set by colleagues	−0.0587 (0.0389)	−0.0412*** (0.0147)
Pace set by targets	−0.0317 (0.0389)	−0.0420*** (0.0158)
Pace set by machines	0.0689 (0.0504)	0.0201 (0.0200)
Pace set by Boss	−0.0221 (0.0397)	−0.0606*** (0.0152)
Number of types of threat/discrimination	−0.0814*** (0.0254)	−0.135*** (0.0130)
Health or safety at risk at work	−0.317*** (0.0466)	−0.359*** (0.0173)
Number of hazards exposed to	−0.0214** (0.00941)	−0.0176*** (0.00405)
Shift work	−0.0965* (0.0539)	−0.0315 (0.0207)
Repetitive tasks	−0.0558 (0.0397)	0.00140 (0.0153)
Monotonous tasks	−0.109*** (0.0395)	−0.158*** (0.0153)
Night shift	0.0443 (0.0538)	−0.00877 (0.0226)
High speed	−0.0827* (0.0429)	−0.0641*** (0.0165)
Observations	3053	26,661
Adj. R^2^	0.282	0.245

Notes:

(1) Robust standard errors in parentheses. ***, ** and * indicate statistical significance at the 1%, 5% and 10% levels, respectively.

(2) Additional controls: an intercept, gender, age, age^2^, income, country (31 dummies), occupation (9 dummies), industry (12 dummies), wages, tenure, hours worked, flexible contract work and firm size, whether the worker can choose speed of work, order of work, or method of work, presence of quality assessment, problem solving required, telework, homework, complex tasks, task rotation, and the need for learning on the job.

**Table 7:** Incentive pay, job satisfaction and job disamenities, BHPS 1998–2008.

	Pooled OLS		Worker-job FE	
Ln wage (2001£)	0.0848*** (0.0142)	0.0877*** (0.0146)	0.100*** (0.0117)	0.102*** (0.0121)
Performance-related pay	−0.0229 (0.0190)	−0.0177 (0.0195)	−0.0129 (0.0166)	−0.0061 (0.0171)
Bonus/profit share	0.0535*** (0.0157)	0.0444** (0.0216)		
Unpaid overtime hours	−0.0045** (0.0022)		−0.0031* (0.0018)	
Commute time (min)		−0.0023*** (0.0004)		−0.0019*** (0.0003)
Bonus/profit share * unpaid overtime hours	0.0057* (0.0030)		0.0064** (0.0025)	
Bonus/profit share * commute time (min)		0.0009 (0.0006)		0.0001* (0.0005)
Observations	52,219	49,895	52,219	49,895
Adj. R^2^	0.063	0.064	0.056	0.058
Number of worker-job matches			1782	1760

Notes:

(1) Robust standard errors in parentheses. ***, ** and * indicate statistical significance at the 1%, 5% and 10% levels, respectively.

(2) OLS models include an intercept and controls for male, age, age^2^, marital status, health status, A-level, diploma, degree or higher, union coverage, large firm (200+), promotion opportunities, employer-funded pension, industry (9 dummies), occupation (9 dummies), and region (11 dummies). Worker-match FE models omit time-invariant controls.

## HIGHLIGHTS

- Group-based performance pay is positively associated with job satisfaction.
- The association is larger for bigger performance payments.
- The association is partly due to the greater organisational loyalty and feelings of fair pay engendered by such payment methods.
- Group-based performance pay mitigates the negative satisfaction effects of exposure to poor working conditions.
- Individual performance pay is not associated with job satisfaction.

## Footnotes

3 One potential concern here, particularly in our single-firm setting, is that individuals receiving group payments may feel obliged to say that they are more loyal to the firm, and perhaps report higher job satisfaction. While we cannot rule this out, it seems less likely to hold in our two other survey settings which are not connected with firms in any way.

4 The ESPP in the UK is a Shareholder Incentive Plan (SIP) that offers tax advantages to those buying and selling shares, together with matched shares from the firm (whereby the firm gives employees a free share for each share they purchase). For details on the Share Plan and the surveys see Bryson and Freeman (2010).

5 The 2010 EWCS lacks the more disaggregated payment-method information available in the previous waves, and so we do not use it here.

6 This is the only satisfaction question in the EWCS.

7 We consider the correlations between these five items via principal components factor analysis with varimax rotation. The items loaded on the two dimensions used to compute the scales described in the text with eigenfactors of 1.17 and 2.72 respectively.

8 Employees were asked to rate themselves on a scale of 1 to 10 in response to the question “*How much do you feel like a co-owner of this company?*” The models are presented in Appendix Table A1.

9 We focus on a subset of these characteristics. The full models are reported as Appendix Table A2.

10 Appendix Table A3 provides sample means for these work conditions.

## Appendix A. Supplementary data

Supplementary data to this article can be found online at http://dx.doi.org/10.1016/j.labeco.2016.09.002.
