# Supplementary material for: Share capitalism and worker wellbeing
Source: Labour Econ. Author manuscript; Available in PMC 2017 Jul 17. (PMC5512719; doi:10.1016/j.labeco.2016.09.002)
Supplement: Appendix [file NIHMS873665-supplement-Appendix.docx]

**On-Line Appendix for “Share Capitalism and Worker Wellbeing” by Alex Bryson, Andrew E. Clark, Richard B. Freeman and Colin P. Green**

Table A1: Job Satisfaction, Share-Plan Membership and Bonuses in ShareCo: the role of co-ownership

|  | OLS | FE | OLS | FE |
| --- | --- | --- | --- | --- |
| Co-ownership | 0.1269***  (0.0092) | 0.1249***  (0.0094) | 0.0398***  (0.0090) | 0.0421***  (0.0093) |
| Member | 0.0930*  (0.0487) | 0.0962*  (0.0510) | 0.0761*  (0.0399) | 0.0671  (0.0426) |
| % Member | 0.0584***  (0.0177) | 0.0653***  (0.0189) | 0.0252*  (0.0149) | 0.0286*  (0.0163) |
| Commission | 0.01553*  (0.0607) | 0.0830  (0.0646) | 0.0433  (0.0478) | 0.0450  (0.0515) |
| Log wage | - | - | -0.0210  (0.0171) | -0.0211  (0.0184) |
| Loyalty | - | - | 0.2183***  (0.0100) | 0.2161***  (0.0101) |
| Fairness | - | - | 0.0379***  (0.0112) | 0.0376***  (0.0115) |
| Adj. R^2^ | 0.15 | 0.16 | 0.43 | 0.42 |

Notes:

(1) The models contain controls described in the notes to Table 1. See the text for details.

(2) Sample N=1,846. Robust standard errors in parentheses. ***, ** and * indicate statistical significance at the 1%, 5% and 10% levels, respectively.

Table A.2: Impact of “Bad” Working Conditions on Satisfaction with Working Conditions among those with and without Share Capitalist Types of Compensation (profit shares or share ownership), Full Set of Job Characteristics.

|  | (1) | (2) |
| --- | --- | --- |
|  | With Share Capitalism | Without Share Capitalism |
|  |  |  |
|  |  |  |
| Commute > 30 Mins | -0.0366 | -0.0250* |
|  | (0.0363) | (0.0142) |
| 10+hours at least once per month | 0.0507 | -0.0249 |
|  | (0.0499) | (0.0205) |
| Work to tight deadlines | -0.0391 | -0.0882*** |
|  | (0.0419) | (0.0178) |
| Pace set by colleagues | -0.0587 | -0.0412*** |
|  | (0.0389) | (0.0147) |
| Pace set by targets | -0.0317 | -0.0420*** |
|  | (0.0389) | (0.0158) |
| Pace set by machines | 0.0689 | 0.0201 |
|  | (0.0504) | (0.0200) |
| Pace set by boss | -0.0221 | -0.0606*** |
|  | (0.0397) | (0.0152) |
| Number of types of threat/discrimination | -0.0814*** | -0.135*** |
|  | (0.0254) | (0.0130) |
| Health or Safety at Risk at Work | -0.317*** | -0.359*** |
|  | (0.0466) | (0.0173) |
| Number of Hazards exposed to | -0.0214** | -0.0176*** |
|  | (0.00941) | (0.00405) |
| Shift work | -0.0965* | -0.0315 |
|  | (0.0539) | (0.0207) |
| Repetitive Tasks | -0.0558 | 0.00140 |
|  | (0.0397) | (0.0153) |
| Monotonous Tasks | -0.109*** | -0.158*** |
|  | (0.0395) | (0.0153) |
| Work at High speed | -0.0827* | -0.0641*** |
|  | (0.0429) | (0.0165) |
| Night Shift | 0.0443 | -0.00877 |
|  | (0.0538) | (0.0226) |
| Tenure | -0.000646 | 0.000479 |
|  | (0.00242) | (0.00102) |
| Hours | -0.000629 | -0.00357*** |
|  | (0.00242) | (0.000836) |
| Flexible Contract | 0.0436 | -0.0419** |
|  | (0.0635) | (0.0186) |
| Worker Chooses Speed | 0.0412 | 0.0565*** |
|  | (0.0479) | (0.0171) |
| Can Change Method of Work | 0.0390 | 0.0317* |
|  | (0.0494) | (0.0179) |
| Can Change Order of Work | 0.0782 | 0.0501*** |
|  | (0.0546) | (0.0178) |
| Quality Assessment | -0.00333 | 0.0756*** |
|  | (0.0442) | (0.0159) |
| Work Involves Problem Solving | -0.0495 | 0.0117 |
|  | (0.0615) | (0.0181) |
| Telework | 0.0773 | 0.0621* |
|  | (0.0739) | (0.0370) |
| Homework | 0.0236 | 0.0330 |
|  | (0.0883) | (0.0338) |
| Job Involves Complex Tasks | -0.0137 | 0.00900 |
|  | (0.0478) | (0.0166) |
| Task Rotation | 0.0219 | 0.0205 |
|  | (0.0384) | (0.0145) |
| Learn New Tasks | 0.0244 | 0.0950*** |
|  | (0.0543) | (0.0173) |
| Constant | 3.167*** | 3.534*** |
|  | (0.400) | (0.110) |
|  |  |  |
| Observations | 3,053 | 26,661 |
| R-squared | 0.282 | 0.245 |

1. Robust standard errors in parentheses. ***, ** and * indicate statistical significance at the 1%, 5% and 10% levels, respectively.
2. Additional controls: gender, age, age^2^, income, country (31 dummies), occupation (9 dummies), industry (12 dummies) and wages.

Table A.3: Summary Statistics on Working Conditions with and without Share Capitalism. EWCS, 2000-2005, Private-Sector Workers.

|  | With Share Capitalism | Without Share Capitalism |
| --- | --- | --- |
| Commute > 30 mins | 0.540 | 0.457 |
| 10+ hours at least once per month | 0.498 | 0.329 |
| Work to tight deadlines | 0.331 | 0.291 |
| Pace set by colleagues | 0.545 | 0.492 |
| Pace set by targets | 0.494 | 0.367 |
| Pace set by machines | 0.217 | 0.220 |
| Pace set by Boss | 0.368 | 0.424 |
| Number of types of threat/discrimination | 0.209 | 0.201 |
| Health or Safety at risk at work | 0.297 | 0.305 |
| Number of hazards exposed to | 1.589 | 1.319 |
| Shift Work | 0.205 | 0.200 |
| Repetitive Tasks | 0.391 | 0.417 |
| Monotonous Tasks | 0.377 | 0.438 |
| Night Shift | 0.220 | 0.182 |
| High Speed | 0.371 | 0.361 |
| **Observations** | **3,053** | **26,661** |
